# Cardiac Tamponade Secondary to Influenza B Infection

**DOI:** 10.7759/cureus.14888

**Published:** 2021-05-07

**Authors:** Praful Schroff, Jacquelyn R Hovey, Cindrel Tharumia Jagadeesan, Vishnu Nagalapuram, Benjamin Chaucer

**Affiliations:** 1 Internal Medicine, University of Alabama at Birmingham (UAB) Montgomery, Montgomery, USA; 2 Internal Medicine, St. Joseph's Hospital and Medical Center/Creighton University, Phoenix, USA

**Keywords:** influenza b, viral pericarditis, cardiac tamponade, pericardial effusion, pericardiocentesis

## Abstract

Every year, Influenza infection contributes to significant morbidity and mortality carrying a huge economic burden. Extra-pulmonary manifestations are increasingly being recognized. We present a 29-year-old woman with acute pericarditis and cardiac tamponade requiring emergent pericardiocentesis secondary to Influenza B infection. Although very rare in relation to Influenza B infection, the pericardial disease can occur during the acute infection or as a post-viral syndrome. Considering pericardial disease in patients with chest pain and any viral infection may facilitate timely diagnosis and prevent unnecessary life-threatening complications.

## Introduction

Pericarditis, or inflammation of the pericardium, is responsible for about 5% of emergency room (ER) visits in the United States (US) [1]. A nationwide analysis estimated the incidence of acute pericarditis in the US to be 5.7 per 100,000 person-years [2]. Even though the US has seen a decline in hospitalizations from pericarditis, the case fatality rate is around 1.8% [2]. Idiopathic and viral etiologies are the most common. Cytomegalovirus, herpes virus, and human immunodeficiency virus (HIV) are the most common viruses causing pericarditis in adults [3,4]. Influenza-related pericarditis and myocarditis are relatively rare. One study found that 2% of all cases where the viral genome could be identified using polymerase chain reaction (PCR) on the pericardial sample were due to the Influenza virus [5]. In this case report, we describe a case of Influenza B pericarditis leading to cardiac tamponade.

## Case presentation

**Figure 1:**
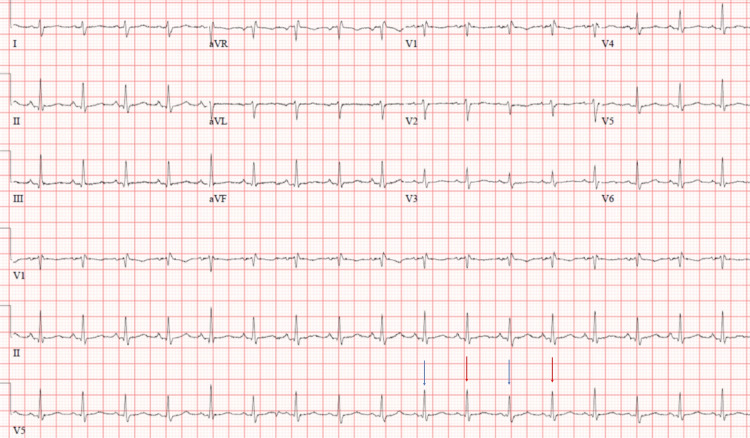
Electrocardiogram revealing electric alternans

**Figure 2:**
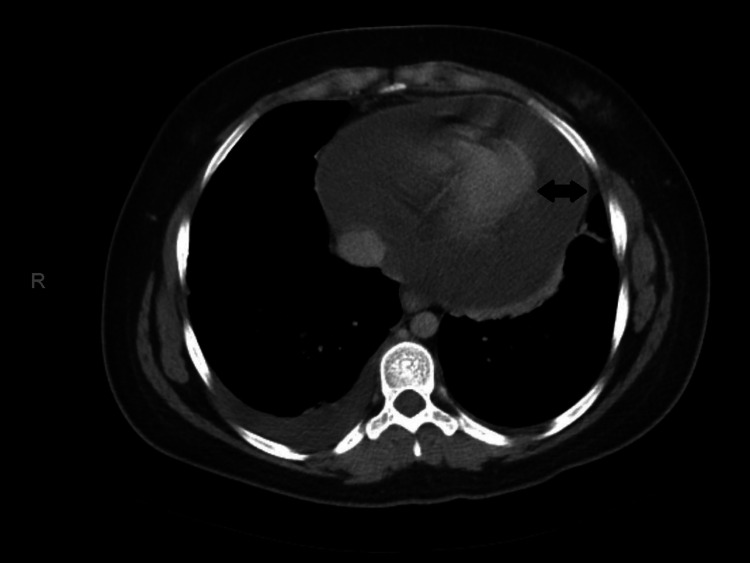
Axial view of the computed tomography of abdomen showing pericardial effusion

A previously healthy 29-year-old Caucasian lady presented to an outside hospital ER during the fall of 2020 with retrosternal chest pain. Her electrocardiogram (EKG) revealed normal sinus rhythm. Serial high sensitivity troponin and d-dimer were normal. A diagnosis of pleurisy was made, and she was discharged home with diclofenac as well as, famotidine for possible gastroesophageal reflux disease. However, her chest pain did not improve, and she went to her primary care physician 15 days later. At this time, she also had a fever, chills, and myalgia. She had not received her vaccination against Influenza that year. She tested positive for Influenza B, and negative for Influenza A and COVID-19. She was given a five-day course of oseltamivir and a seven-day-course of azithromycin. However, her chest pain continued to worsen, and she presented to the same ER three days later with severe chest pain, abdominal discomfort, nausea, and vomiting. Her chest pain worsened when lying flat and was associated with shortness of breath, especially with exertion. She denied any lightheadedness, syncope, or palpitations. She was visibly uncomfortable, tachycardic with a heart rate of 134 beats per minute, and had a blood pressure of 110/70 mm Hg. She did not have a pericardial rub but did have muffled heart sounds. There was no jugular venous distension, pedal edema, or pulsus paradoxus. EKG showed electrical alternans (Figure 1). Chest radiograph showed cardiomegaly, and computed tomography (CT) of the abdomen showed a large pericardial effusion (maximum width: 2.75 cm) (Figure 2). She was emergently transferred to our hospital ER for further management.

**Figure 3:**
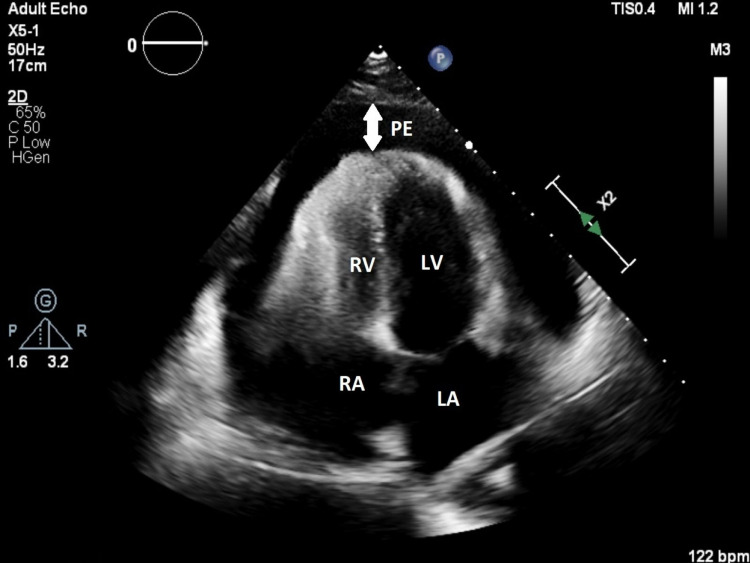
Transthoracic echocardiogram (apical view) revealing pericardial effusion LA - left atrium; RA - right atrium; LV - left ventricle; RV - right ventricle; PE - pericardial effusion

At presentation, a bedside ultrasound showed a large pericardial effusion with right ventricular collapse later confirmed with transthoracic echocardiogram (TTE) (Figure 3). She was started on intravenous fluids to maintain adequate preload. She underwent emergent pericardiocentesis with 920 milliliter of serous fluid removed. Fluid studies showed increased mononuclear cells representing an inflammatory process. Bacterial, mycobacterial, viral and fungal studies of the fluid were negative. Influenza B PCR test was not done in the pericardial fluid. On admission, her erythrocyte sedimentation rate was 63 millimeters per hour, C- reactive protein 18.52 milligram per deciliter. Blood urea and troponin were normal and HIV, anti-nuclear antibodies (ANA) screen, and drug screen were negative. Chest pain improved over the next day and effusion had decreased in size. She was discharged on colchicine and ibuprofen. An outpatient TTE at the cardiologist office one week later showed no re-accumulation of fluid. She was also seen in our primary care clinic 10 days after discharge, by which time her symptoms had resolved and she was also vaccinated for Influenza.

## Discussion

The pericardium is a sac-like structure that surrounds the heart and consists of two layers, a visceral layer which is adherent to the cardiac epithelium and a fibrous parietal layer composed of collagen and elastin. Between these layers lies a potential space, which may become inflamed, causing pericarditis, or if filled with blood or fluid, a pericardial effusion. This in turn can compress the heart itself, especially the right ventricle, and impair diastolic function; referred to as cardiac tamponade. To diagnose acute pericarditis, at least two of the following should be met: 1) chest pain; 2) pericardial rub; 3) EKG changes, and 4) new or worsening pericardial effusion [6]. Pericarditis and its complications can be the result of infection, uremia, neoplasm, cardiac injury/trauma, systemic autoimmune disease, mediastinal radiation, etc. [7]. Of these inflammatory etiologies, the most common causes of acute pericarditis are idiopathic or viral [8]. The Influenza virus, in particular, is a virus occasionally associated with pericarditis and resulting cardiac tamponade. Influenza has been noted to cause both myocarditis, inflammation of the cardiac muscle, as well as pericarditis. Such cases are generally mild and clinically insignificant. However, Influenza has rarely been associated with significant myocardial or pericardial disease resulting in cardiac tamponade, hemodynamic instability, and mortality [9,10].

The Influenza virus has two main subtypes, Influenza A and Influenza B. Though both subtypes can be implicated in epidemic seasonal infections, with a total of three to five million cases and over 300,000 deaths annually, Influenza B is generally considered a milder form of flu and only rarely causes non-respiratory complications when compared with Influenza A [11-13]. Additionally, Influenza B only accounts for approximately 0.08% of circulatory and/or respiratory-related hospitalizations [14]. In most reported cases of severe pericarditis or cardiac tamponade secondary to Influenza, it is Influenza A that is implicated [15-17].

There are, however, some recent reports of significant cardiac manifestations of Influenza B. Arfaras-Melainis et al. [18] reported a patient who tested positive for Influenza B who returned to the ER, five days after prior discharge, with worsening dyspnea with hypotension and tachycardia. A bedside point of care ultrasound (POCUS) was performed, along with TTE, which showed pericardial effusion and cardiac tamponade with right ventricular diastolic collapse. Spoto et al. [13] likewise presented a patient with a recent flu-like illness who subsequently presented to the ER with increased dyspnea and was diagnosed to have a large pericardial effusion. PCR of tracheal aspirate confirmed Influenza B infection.

Roto et al. [19] reported another interesting case of a previously healthy patient with Influenza B who presented to the ER acutely ill, with refractory hypotension, hypothermia, tachycardia, altered mental status, and severe lactic acidosis. A bedside POCUS was performed and revealed a large pericardial effusion and cardiac tamponade. Such cases indicate the necessity to assess for pericardial effusion due to Influenza B with a high clinical index of suspicion given its low incidence.

Ultimately, our patient’s illness duration and progression to cardiac tamponade could have been significantly shortened if she had been evaluated for pericardial effusion at the prior ER and/or clinic visits. Also, timely initiation of oseltamivir and vaccination against Influenza remains the cornerstone in reducing its morbidity and mortality. Not all pericardial effusions produce classic clinical and EKG findings, and therefore, such a diagnosis should be strongly considered if there is a history of Influenza infection with worsening chest pain or increasing dyspnea. Bedside ultrasound can be a crucial modality for a quicker evaluation and aid in preventing mortality in such instances of pericarditis and cardiac tamponade secondary to Influenza B.

## Conclusions

Pericarditis, with or without cardiac tamponade, is often associated with viral infections. Although a well-recognized manifestation or complication of Influenza A, pericarditis and resultant cardiac tamponade has rarely been associated with Influenza B infection. It is important, therefore, that worsening chest pain and/or dyspnea during acute or with recent Influenza B infection be further evaluated to assess for pericarditis and cardiac tamponade and treated accordingly to prevent associated morbidity and mortality.
